# Bony Tumor of the Upper Jaw—Amputation

**Published:** 1853-04

**Authors:** 


### ARTICLE XVI.

### *Bony Tumor of the Upper Jaw—Amputation.*

Mr. Hancock related the following case to the *Medical Society* of London:—T. C., aged twenty-two, the son of a respectable farmer, residing at Bicker, in Lincolnshire, was admitted into the Charing Cross Hospital, October 1st, 1840, suffering from a bony tumor connected with the superior maxilla. His friends state, that, when about a year and a half old, he was found on the floor, supposed to be in a fit; when taken up, he appeared to be stunned, and the right side was slightly affected. No notice was, however, taken of it, and he quickly recovered from the immediate symptoms, but always afterwards complained of pain in the right side of the face, as far back as the ear. About two years after the accident, that side of the face was first noticed to protrude more than the other, and since then it has gradually enlarged, but more rapidly within the last three years. He has always complained of pain in the tumor, more particularly after it had been touched or examined. His general health has been pretty good, but he has not followed any employment on account of his appearance. He is rather deaf, but his friends do not think the deafness has increased with the enlargement of the bone, for which he has not been subjected to any treatment. He is much disfigured by the disease, a large tumor projects more than two inches forwards, and to the left side, invading the nose, which it has flattened and spread, and turned completely to the left, so that the openings of the nostril, instead of being directed downwards, and in the medial line, are turned to the left side of the face, and directed forwards; the right nostril is completely filled by the tumor, except at its upper part, where, with a little difficulty, a curved probe may be carried over it, but the septum naris is touched, and pushed into the left nostril so completely, that a probe cannot be introduced between the septum and tumor along the floor of the nostril. The hard palate of the right side is thickened, and projects into the mouth, as it also does on the left side. The tumor not only extends forwards, but also towards the right side, generally implicating the malar bone, which is much thickened and enlarged, as is also the frontal bone at its external angle; the zygomatic process remains natural; the right eye appears raised; the disease does not extend backwards towards the fauces, neither are the motions of the lower jaw at all impeded. The right side of his head is larger than the left, and the surface of the frontal bone presents prominences; thickening having taken place in those situations; his general health is good; he sleeps and eats well, his pulse is regular and quiet. A consultation having been held upon the case, and it having been considered non-malignant, and a proper one for operation; I, on Thursday last, removed the parts which are now before the Society. The patient being secured in a firm chair, so that he could not slide away, the first incisor tooth of the left side was extracted, and I commenced the first incision below the tendon of the orbicularis palpebrarum muscle, and carried it downwards by the ala of the nose, directly through the upper lip, taking care to keep the point of the knife steadily against the bone, so that the soft parts were completely divided by the one cut; a second incision was then made inwards from the lobe of the ear to the ala of the nose, and a third from about an inch above the middle of the zygoma, directly downwards to the second incision. The flaps thus made were then reflected, the one downwards and the other upwards, the dissection of the latter being continued so as to expose the external angle of the frontal bone, which was found to be much enlarged and thickened, and the floor of the orbit, the attachment of the inferior oblique muscle of the eye being divided. The parts being thus fairly exposed, a cut was made with a saw obliquely downwards and backwards, from about a quarter of an inch above the root of the zygoma into the spheno-maxillary fissure; the ala of the nose was next reflected, and the ascending or nasal process of the superior maxillary bone cut through by strong bone forceps, as was the floor of the orbit; with a strong knife the masseter muscle was next detached from the zygoma, which was cut through by the forceps, and the patient was allowed a short time to recover from the chloroform, under the influence of which he had been placed for these more superficial steps of the operation; a very short interval sufficed. One blade of the bone forceps was then carried firmly backwards into the right nostril, the other blade being placed on the hard palate of the left side, and the palate was thus divided obliquely downwards and to the left, as I was desirous, if possible, to preserve the septum naris attached, and, at the same time to remove the whole of the thickened palate. It required some little power to do this, but the forceps went through without much difficulty, although the parts were considerably thickened. As the bone still remained firm, and I could not detach it with my finger and thumb, I concluded that the malar bone had not been completely divided by the saw, and accordingly used the bone forceps in that situation, after which I could with ease depress the bone, and complete the operation by cutting through the soft palate with a blunt-pointed bistoury. The parts removed were the malar, the superior maxillary, and the palate bones. The arteries were secured as quickly as possible, but some difficulty was experienced in securing the infraorbital, which had shrunk back into its bony canal, and could not be tied; the bleeding, however, was arrested by plugging up the canal with lint, but the patient lost a large quantity of blood, and became very faint whilst the sutures were being applied; the flaps were very carefully brought together, and the patient placed in bed. The chloroform was administered to diminish the pain of the more superficial cuts; it was not only discontinued, but the patient allowed to become perfectly conscious before the palates, either hard or soft, were meddled with, lest any blood should enter the glottis and do mischief; he bore the whole operation with the greatest steadiness and fortitude. In the course of the evening he vomited twice, and brought up a large quantity of blood, which he had swallowed during the operation; he passed a restless night, notwithstanding he had an opiate draught; towards morning he became more quiet, and went to sleep for two or three hours. He has gone on since extremely well up to this time; the wounds in the face have almost entirely healed by the first intention. I have sawed through the jaw and tumor, to expose the structure of the latter. It appears to me to have originated from the antrum, and to have caused absorption of the body of the superior maxillary bone, excepting the hard palate and alveolar processes, and its malar process, which, with the malar bone, are much thickened, enlarged, and very hard, whilst the tumor itself is of a softer texture, very like the cancellated structure of bone. The parts removed weighed seven ounces and one drachm.

